# The effects of hearing loss on neural processing and plasticity

**DOI:** 10.3389/fnsys.2015.00035

**Published:** 2015-03-06

**Authors:** Arthur Wingfield, Jonathan E. Peelle

**Affiliations:** ^1^Volen National Center for Complex Systems, Brandeis UniversityWaltham, MA, USA; ^2^Department of Otolaryngology, Washington University in St. LouisSt. Louis, MO, USA

**Keywords:** hearing loss, auditory cortex, cognition, aging, listening effort

Hearing loss—ranging from mild to severe—afflicts large numbers of individuals of all ages. It is estimated that 40–50% of adults over the age of 65 years have some degree of significant hearing loss, with this figure rising to 83% of those over the age of 70 (Cruickshanks et al., 1998). This makes hearing loss the third most prevalent chronic medical condition among older adults after arthritis and hypertension (Lethbridge-Cejku et al., 2004). Recent years have seen increasing appreciation for the downstream consequences of reduced hearing acuity, even when perception itself has been successful. In the case of speech, these consequences include negative effects of perceptual effort on encoding what has been heard in memory (Rabbitt, 1991; Surprenant, 1999; Pichora-Fuller, 2003; McCoy et al., 2005; Cousins et al., 2014) and comprehension of sentences whose processing is resource-demanding because of complex syntax (Wingfield et al., 2006). Beyond these short-term effects, there also appear to be small but statistically significant correlations between hearing acuity and the appearance of all-cause dementia (Gates et al., 2011; Lin et al., 2011b) and performance on standardized cognitive tests in non-demented individuals (Lin et al., 2011a). Strikingly, the relationship between hearing acuity and cognitive ability holds even when adjusted for sex, age, education, diabetes, smoking history, and hypertension (Lin, 2011; Lin et al., 2011a; Humes et al., 2013a).

The effects of impaired hearing thus goes beyond difficulty in speech recognition. Speech comprehension in the face of mild-to-moderate hearing loss modifies patterns of neural activation in BOLD imaging, and analyses of structural MRI images have shown that poor hearing acuity is associated with reduced gray matter volume in auditory cortex (Peelle et al., 2011; Eckert et al., 2012; Lin et al., 2014). Findings such as these indicate a biological link between sensory stimulation and cortical integrity, consistent with animal models demonstrating neural reorganization when sensory input is disrupted. In humans, these effects on auditory cortex may have cascading influences throughout the hierarchical set of regions involved in speech processing (Davis and Johnsrude, 2003; Rauschecker and Scott, 2009; Peelle et al., 2010).

Understanding sensory-cognitive interactions represents an important research challenge, especially when changes in hearing acuity are compounded by declines in working memory resources and executive function that often occur in adult aging. One must also note claims of an increase in hearing loss among young adults (Shargorodsky et al., 2010), many of whom remain unaware of their hearing loss and the consequences of perceptual effort on cognitive performance (Widen et al., 2009; Le Prell et al., 2011). At the level of remediation, surgically placed cochlear implants have seen increasing use, to include use with older adults, when hearing acuity has declined to a point where standard hearing aids no longer yield significant benefit (Dillon et al., 2013). This emerging technology will call increasingly on the translational potential of basic research in auditory physiology currently active in human and animal studies.

This research topic presents a collection of original articles that explore the cognitive and neural consequences of hearing loss, including basic processes carried out in the auditory periphery, computations in subcortical nuclei and primary auditory cortex, and higher-level processes such as those involved in human speech perception. Together, these articles form a compelling body of work demonstrating numerous ways in which brain structure, neural function, and behavior are impacted by hearing loss.

We begin with seven review and theory articles. Rönnberg and coauthors offer a timely update of the Ease of Language Understanding (ELU) model in which they stress the importance of working memory for online spoken language processing, especially under poor listening conditions (Rönnberg et al., 2013). Heald and Nusbaum (2014) continue this theme, arguing that even early-stage speech recognition is an attentionally-guided active process and not as automatic as some have suggested. Review articles by Guediche et al. (2014) and by Keating and King (2013) stress the flexibility in the perceptual system that allows for adaptation to auditory perturbations. Eggermont (2013) and by Butler and Lomber (2013) focus primarily on animal models to explore effects of experience on auditory processing, while Bharadwaj et al. (2014) review human and animals studies demonstrating that precision in temporal coding may be poor even when hearing thresholds are normal. Taken together, these papers emphasize the view that auditory detection thresholds give only a limited picture of auditory and auditory-cortical processing.

Additional evidence bearing on plasticity and development appears in six research articles using animal models. Gay et al. (2014) and Kang et al. (2014) explore mechanisms underlying interactions between early conductive hearing loss and effects on detection tasks in adulthood, while Kamal et al. (2013) focus on impact and reversibility of noise exposure effects in auditory cortex. Huetz et al. (2014) examine functional modification to cortical cells in response to moderate hearing loss. Henry et al. (2014) report effects of noise-induced sensorineural hearing loss on complex temporal coding, and Kral et al. (2013) examine the implications of hemisphere asymmetries in cortical adaptation to unilateral hearing loss in development.

Studies in human listeners reveal many of the same aspects of plasticity in the perceptual system as seen in animal models. Avivi-Reich et al. (2014) illustrate the dynamic interaction between bottom-up input and top-down cognitive factors when older adults are challenged by listening to a target speaker in a background of multiple speakers and when listening in a second language. Mishra et al. (2013) continue this theme with an emphasis on the role of selective attention when listening to speech in noise. Humes et al. (2013b) examine individual difference factors that influence successful speech comprehension beyond peripheral hearing acuity. The value of in-depth studies of a single individual is illustrated by Firszt et al. (2013) who report neural and performance changes in an adult patient following successful surgery for a congenital unilateral hearing loss. Anderson et al. (2013) offer additional evidence bearing on plasticity in the sensory-cognitive system in a study of compensatory training through directed attention in hearing impaired older adults. McGettigan et al. (2014) address learning-related changes in speech recognition using noise-vocoded speech to simulate the acoustic input available from a cochlear implant. Finally, Ihlefeld et al. (2014) focus their research article on factors relating to cochlear implant recipients' decrements in the use of interaural time differences for localizing sound sources in space.

Considerable advances have been made using a number of human brain imaging techniques, as illustrated by a final eight articles in this collection that have examined effects of hearing loss using diffusion tensor imaging (DTI) to assess white matter integrity (Rachakonda et al., 2014), functional MRI to reveal patterns of neural reorganization and compensatory cognitive control with hearing loss and aging (Erb and Obleser, 2013; Husain et al., 2014), patterns of neural responses using electroencephalograph (EEG) recordings from scalp electrodes (Becker et al., 2013; Campbell and Sharma, 2013; Catz and Noreña, 2013; Tremblay et al., 2014) and magnetoencephalography (MEG) to examine contributory effects of reduced inhibitory control in older adults with hearing impairment (Alain, 2014).

Together, these collected articles reflect a valuable sample of current approaches to our understanding of the effects of hearing loss on neural and perceptual processing. A theme that emerges from both the human and animal studies in this collection is that of an adaptive plasticity in the sensory, perceptual and cognitive systems that regulates performance in the face of often seriously degraded input. Challenges for future research include better understanding the link between neural consequences of hearing loss and other modifications of acoustic input (Van Engen and Peelle, 2014) and a more direct linking of hearing ability, brain structure, neural function, and behavior.

## Conflict of interest statement

The authors declare that the research was conducted in the absence of any commercial or financial relationships that could be construed as a potential conflict of interest.
